# Identification of Upper Respiratory Tract Pathogens Using Electrochemical Detection on an Oligonucleotide Microarray

**DOI:** 10.1371/journal.pone.0000924

**Published:** 2007-09-26

**Authors:** Michael J. Lodes, Dominic Suciu, Jodi L. Wilmoth, Marty Ross, Sandra Munro, Kim Dix, Karen Bernards, Axel G. Stöver, Miguel Quintana, Naomi Iihoshi, Wanda J. Lyon, David L. Danley, Andrew McShea

**Affiliations:** 1 CombiMatrix Corporation, Mukilteo, Washington, United States of America; 2 United States Army Center for Health Promotion and Preventive Medicine-West, Fort Lewis, Washington, United States of America; 3 Air Force Research Laboratory/Human Effectiveness Directorate, Applied Biotechnology Branch, Wright-Patterson Air Force Base, Ohio, United States of America; Medical University of South Carolina, United States of America

## Abstract

Bacterial and viral upper respiratory infections (URI) produce highly variable clinical symptoms that cannot be used to identify the etiologic agent. Proper treatment, however, depends on correct identification of the pathogen involved as antibiotics provide little or no benefit with viral infections. Here we describe a rapid and sensitive genotyping assay and microarray for URI identification using standard amplification and hybridization techniques, with electrochemical detection (ECD) on a semiconductor-based oligonucleotide microarray. The assay was developed to detect four bacterial pathogens (*Bordetella pertussis, Streptococcus pyogenes, Chlamydia pneumoniae and Mycoplasma pneumoniae*) and 9 viral pathogens (adenovirus 4, coronavirus OC43, 229E and HK, influenza A and B, parainfluinza types 1, 2, and 3 and respiratory syncytial virus. This new platform forms the basis for a fully automated diagnostics system that is very flexible and can be customized to suit different or additional pathogens. Multiple probes on a flexible platform allow one to test probes empirically and then select highly reactive probes for further iterative evaluation. Because ECD uses an enzymatic reaction to create electrical signals that can be read directly from the array, there is no need for image analysis or for expensive and delicate optical scanning equipment. We show assay sensitivity and specificity that are excellent for a multiplexed format.

## Introduction

Upper respiratory tract infections (URI) can be caused by a variety of viruses, including rhinovirus, coronavirus, influenza A and B, parainfluenza, respiratory syncytial virus, adenovirus, metapneumovirus, and enterovirus; and by bacteria, including *Chlamydia pneumoniae, Mycoplasma pneumoniae, Streptococcus pyogenes, S. pneumoniae, Bordetella pertussis, and Haemophilus influenzae* [1], [2], [3]. Clinical symptoms of URI are highly variable and cannot be used to identify the etiologic agent or agents. Although the majority of URI are caused by viruses (69%; [3]), antibiotics are widely prescribed to treat symptoms. Antibiotics, which are of little or no benefit for viral infections, can contribute to the emergence and spread of resistant bacteria and to higher costs for health care [4], [5], [6].

The rapid identification of certain viruses and bacteria is necessary for proper treatment of infection. Patients with a history of rheumatic fever (*Streptococcus*), patients with bacterial or viral infections for which specific therapies are available, and patients whose URI symptoms persist for over two weeks, can benefit from rapid detection. Rapid and accurate identification of URI agents at the point of care can reduce the inappropriate prescription of antibiotics and help identify problematic infections early.

Traditional methods for the identification of URI pathogens, including culture and serology, are effective but are labor intensive and may require highly trained personnel and days to weeks to complete. Many rapid serological tests are not sensitive or specific enough to detect all infections. Enzyme immunoassays (EIA) generally show sensitivities of 85% and direct immunofluorescence (DIF) assays show sensitivities of approximately 60 to 80% [7]. Although viral culture is generally accepted as the gold standard for diagnosis, PCR can be more sensitive [8]. In one study, rhinovirus culture detected infection in 40% of patients while rhinovirus PCR detected infection in 51.5% of patients [3]. Similarly, an influenza virus surveillance study showed that 18% of samples were positive by culture and 28% were positive by TaqMan-PCR [9].

Because of their speed, specificity and sensitivity, genomic assays are complementary to serological assays and especially useful for identifying an unknown specimen where antibodies are not specific enough to differentiate closely related groups [10], [11], [12], [13], [14], [15], [16], [17], [18], [19], [20], [21], [22]. Reverse transcription-polymerase chain reaction (RT-PCR) is widely used for identifying virus and bacteria; however, a positive amplification is often verified by subsequent procedures that provide sequence information [8], [23], [24]. By using multiple probes against a single pathogen coupled with the proper amplification scheme, microarrays can serve as valuable tools for viral discovery, detection, genotyping and sequencing [13], [14], [21], [25], [26], [27], [28], [29], [30], [31], [32], [33].

While traditional assays for pathogen detection and typing represent the gold standard, they alone, cannot meet the future needs for rapid, sensitive, specific and simple methods. The majority of these approaches do not provide sequence information, utilize expensive equipment that cannot be easily transported, require a high level of expertise to operate equipment, require extended periods of time, or are not easily modified. Here we describe a rapid and sensitive genotyping assay and array for upper respiratory pathogen identification using standard amplification and hybridization techniques and electrochemical detection (ECD) on a semiconductor-based oligonucleotide microarray. This platform was particularly suitable for developing this assay because it supports custom probe synthesis, which permits oligonucleotides of interest to be tested empirically. Multiple, highly reactive probes that are specific for a particular organism can then be put on the array for further iterative evaluation. Content of the final assay can also be customized to suit diagnostic needs in different geographic areas. Finally, the ability to use electrochemical detection with semiconductor microchips eliminates the need for expensive and delicate optical scanning equipment [34], [35], [36]. Scanning of microarrays is typically labor intensive and a significant source of system noise that complicates the use of microarrays significantly; and has hampered the transition of these technologies into high throughput clinical scenarios by adding undue instrumentation cost and complexity. The ECD method of deriving signal from a microarray is extremely simple and provides a clear path to making microarray processing truly hands off throughout the entire process.

## Results

### URI pathogen genotyping assay

The assay was designed to identify 10 upper respiratory pathogens, with subtype identification in some cases (coronavirus and parainfluenza virus). We utilized multiple probes for each pathogen (approximately 30 to 100) that were designed from conserved regions of the respective genomes. Approximately 850 unique URI probes were included on the array with 12 to 13 replicates for each probe. The assumption for this methodology is that enough unique probes will produce specific signal in the presence of the labeled PCR products to result in a positive call. Multiple probes allow for genetic variability, especially with viral genomes, and also provide sequence information. In principle it is straightforward to substitute other organisms in these assays by synthesizing arrays with alternate probe specificity and alternate primer sets.

### Amplification and labeling

**Figure 1:**
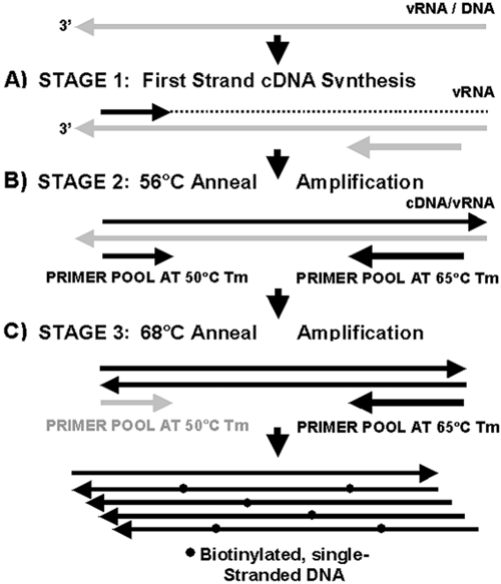
Strategy for amplification and labeling of pathogen-specific target. The one-tube RT-PCR reaction includes three stages: A) Reverse transcription reaction to produce first strand cDNA for viral targets (vRNA); B) Low temperature (56°C), exponential amplification of target and C) High temperature (68°C), linear amplification to product single-strand, biotin-labeled target. Target is labeled by incorporation of biotin-14-dCTP during amplification or by the use of biotinylated reverse primers.

One-tube RT-PCR was used for target amplification and labeling because of its sensitivity and its application to both DNA and RNA targets (Fig. 1). Our PCR primers flanked conserved regions that had complementary sequences to the probes on the array. The combination of microarrays with RT-PCR allowed for the detection of specific signal from a complex reaction mix containing background NA. The PCR amplification/labeling reactions took approximately 3.5 hrs with a peltier thermal cycler; however the reaction time was reduced to less than two hours using a LightCycler (Roche). Single-strand target was produced by adjusting the melting temperature of the forward and reverse primer pools to approximately 50°C and 65°C respectively. This 15°C temperature differential between primer sets allowed for a 50°C reverse transcription step followed by exponential amplification at 56°C. Increasing the annealing temperature of the reaction to 68°C, with 30 additional rounds of amplification, resulted in a predominantly one-way reaction favoring reverse primer extension. Biotin labeling occurred during amplification by incorporation of biotinylated dCTP/dATP in the reaction mix. Biotinylated reverse primers were also employed as an alternate method for biotin labeling.

**Figure 2:**
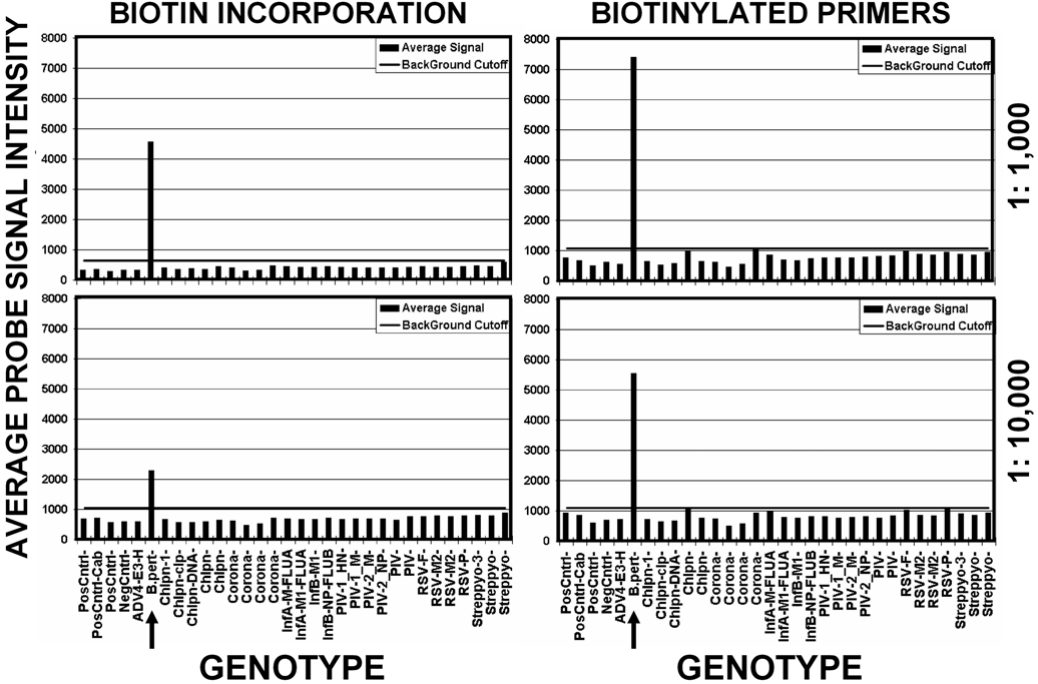
Comparison of assay sensitivity with target labeled by biotin-incorporation and biotinylated primers. *B. pertussis* (*B.pert*, arrow) genomic DNA at 15 ng/ul was diluted to 1∶1,000 (top panels) and 1∶10,000 (bottom panels) and labeled either by biotin-incorporation (left panels) or with biotinylated reverse primers (right panels). The average of type-specific probe group signals in picoamps is shown at the left and identity of pathogen type-specific probe groups is shown below panels. The horizontal bar indicates the assay cutoff, which was determined by the mean value of negative control probes plus 3 standard deviations of the mean.

We compared the sensitivity and specificity of biotin labeling by incorporation during amplification versus biotinylated reverse primers (Fig. 2). Although using biotinylated reverse primers increased assay sensitivity, nonspecific signal increased due to binding of free biotinylated oligonucleotides to specific probes. This background could be eliminated during analysis by removing approximately 2% of the high and low signal for each genotype category prior to averaging.

### Hybridization and electrochemical detection

**Figure 3:**
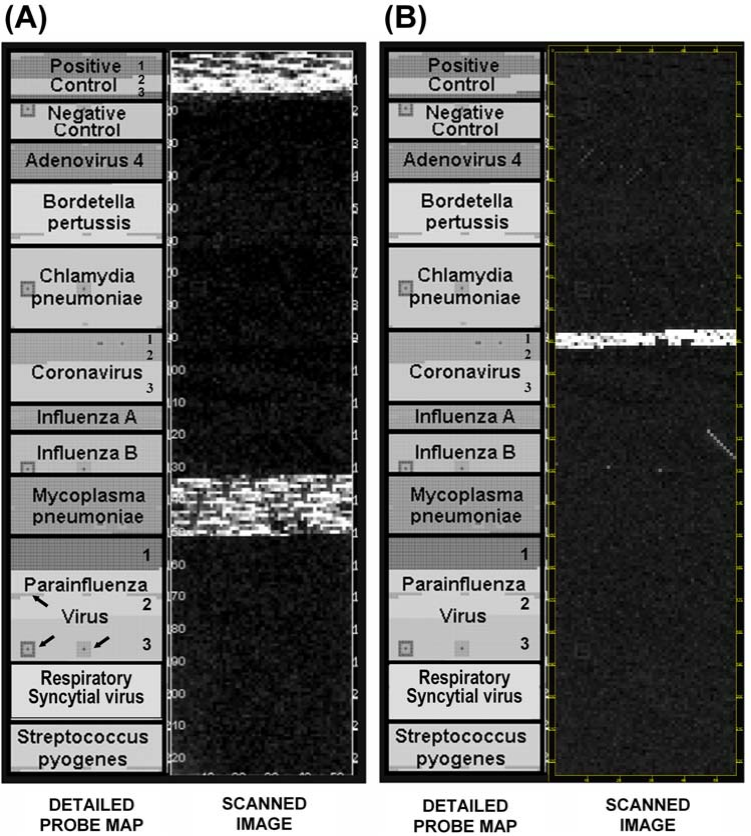
Pseudoimage of raw data from scanned chips illustrating *Mycoplasma pneumoniae* (A) and coronavirus 229E (B) detection. The left panels (Probe Map) show the position of all pathogen-specific probes and include 3 sub-type probes for coronavirus and parainfluenza virus. Geometric shapes indicated by arrows are areas on the microarray dedicated to array quality control probes. Positive control target was included only with *M. pneumoniae* (A). Coronavirus probes are divided into three sections on the array: 1) Type 1, 2) Type 2, and 3) Type 3.

While the microarray can be used with fluorescence detection, we used electrochemical detection (ECD), which relies on a redox reaction to generate electrical current (pA) on the array for measurement. Pseudo-images of two hybridized targets are shown in Figure 3A (*M. pneumoniae*) and B (coronavirus 229E). These images are a representation of hybridization signals (i.e. current) derived from the raw data. This is in contrast to conventional microarray technology where the data are derived from the image and subsequently analyzed. Signal is predominately seen in the respective block of specific probes for each organism with scattered background signal. Variation of signal intensity within a pathogen-specific block of probes resulted from: 1) variable biotin labeling due to amplicon size and GC content; 2) variation in probe-target interaction due to genomic variability; and 3) predictable and un-predictable nucleotide base-pairing characteristics of probes. To overcome these issues, we determined a specific genotype based on the average signal intensity of all pathogen-specific probes, which includes 12 to 13 replicates of each probe. This was done rapidly using an Excel macro to generate bar graphs to identify the most likely candidate(s).

### URI assay validation

**Figure 4:**
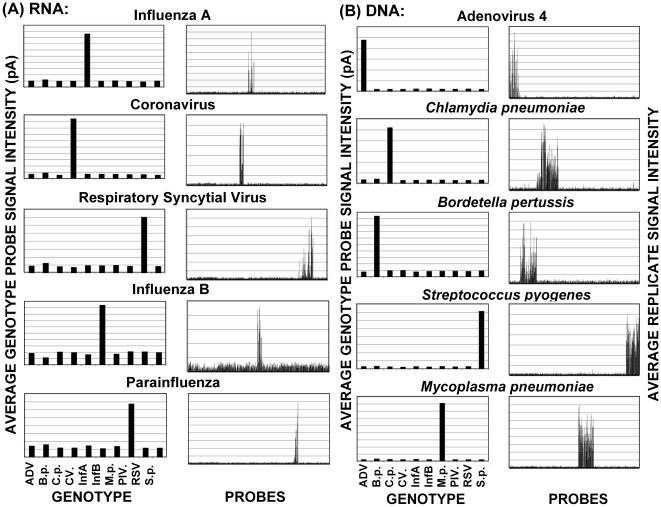
URI chip validation studies showing average RNA genome (A) and DNA genome (B) target signal intensity for the 10 upper respiratory pathogens studied. The left panels (GENOTYPE) show the average genotype-specific probe signal in picoamps that is used to determine a genome identity. The right panels (PROBES) illustrate the average probe signal intensities of 12 replicates for each probe and also illustrate signal specificity for both positive and negative probes. Pathogens are ADV (adenovirus 4), *B.p*. (*Bordetella pertussis*), *C.p*. (*Chlamydia pneumoniae*), CV (coronavirus), InfA (influenza A), InfB (influenza B), *M.p*. (*Mycoplasma pneumoniae*), PIV (parainfluenza virus), RSV (respiratory syncytial virus), and *S.p*. (*Streptococcus pyogenes*) at nucleic acid concentrations described in Table 2.

Results of array validation studies are shown in Figure 4. The prototype URI array contained probes representing two to six amplicons per organism (see Table 1) and two primer pools consisting of 18 primer pairs for RNA targets and 19 primer pairs for DNA targets (pools 1A and 2A respectively). Both pools contained two primer pairs for the Arabidopsis positive control (Cont.) spike-in nucleic acids for both DNA and RNA genome targets (spike-ins were predominately used for unknown samples and not for validation). Assays were designed to genotype each of the 10 URI pathogens with coronaviruses and parainfluenza viruses subdivided into three subtypes. Each of the 10 target genomes was successfully identified with little or no background cross-reactivity (Figs 4A and B).

**Table 1:** Primer pools, amplicons and probes for the URI assay.

Pool	Target	Forward	Reverse	Gene	Amplicon	A	B	C	Probes
1	*B. pertussis*	Bp_For_1	Bp_Rev_1	LysR TR	427	*			8
1	*"*	Bp_For_2	Bp_Rev_2	ABC T	476	*	*	*	18
1	*"*	Bp_For_3	Bp_Rev_3	ADH	769	*			14
1	*"*	Bp_For_4	Bp_Rev_4	LysR TR	569	*			21
1	*"*	Bp_For_5	Bp_Rev_5	tfdA	552	*			10
1	*"*	Bp_For_6	Bp_Rev_6	CAIB	534	*	*		15
1	*M. pneumoniae*	Mp_For_1	Mp_Rev_1	dnaK	564	*			29
1	*"*	Mp_For_2	Mp_Rev_2	pdhA	284	*	*	*	18
1	*"*	Mp_For_3	Mp_Rev_3	tuf	604	*	*		33
1	*S. pyogenes*	Sp_For_1	Sp_Rev_1	3-oxoacyl	510	*			19
1	*"*	Sp_For_2	Sp_Rev_2	23S rRNA	463	*	*		26
1	*"*	Sp_For_3	Sp_Rev_3	ABC T	421	*	*	*	24
1	*C. pneumoniae*	Cp_For_1	Cp_Rev_1	clp endop	491	*			23
1	*"*	Cp_For_2	Cp_Rev_2	HB-2	528	*	*	*	25
1	*"*	Cp_For_3	Cp_Rev_3	29 kDa lip	475	*			23
1	*"*	Cp_For_4	Cp_Rev_4	RNA pol S	526	*			22
1	*"*	Cp_For_5	Cp_Rev_5	R-Isomer	526	*	*		20
1	Adenovirus 4	Adv_For_1	Adv_Rev_1	gp 12	531	*	*		55
1	*"*	Adv_For_2	Adv_Rev_2	gp 12	606	*	*	*	**
2	Coronavirus 229E	CV229E_F_1	CV229E_R_1	Matrix	299	*	*		20
2	Coronavirus HK	CV HK_F_1	CV HK_R_1	HE	767	*	*		22
2	Coronavirus OC43	CVOC43_F_1	CVOC43_R_1	HE	528	*			28
2	"	CVOC43_F_2	CVOC43_R_2	N	507	*	*		33
2	Influenza A	InfA_For_1	InfA_Rev_1	Matrix	500	*	*		20
2	"	InfA_For_2	InfA_Rev_2	Matrix	339	*			13
2	Influenza B	InfB_For_1	InfB_Rev_1	NP	516	*	*		29
2	"	InfB_For_2	InfB_Rev_2	Matrix	506	*			28
2	PIV 1	PIV1_For_1	PIV1_Rev_1	Matrix	488	*	*		28
2	"	PIV1_For_2	PIV1_Rev_2	HN	532	*			23
2	PIV 2	PIV2_For_1	PIV2_Rev_1	Matrix	576	*			28
2	"	PIV2_For_2	PIV2_Rev_2	NP	592	*	*		34
2	PIV 3	PIV3_For_1	PIV3_Rev_1	Matrix	840	*	*		35
2	"	PIV3_For_2	PIV3_Rev_2	HA	517	*			24
2	RSV	RSV_For_1	RSV_Rev_1	Matrix 2	440	*			18
2	"	RSV_For_2	RSV_Rev_2	Matrix 2	496	*			22
2	"	RSV_For_3	RSV_Rev_3	Phos	412	*			23
2	"	RSV_For_4	RSV_Rev_4	Fusion	474	*	*		18
Cont.	Arabidopsis	RCA For	RCA Rev	RCA	508	*	*	*	32
Cont.	"	Cab For	Cab Rev	caB	501	*	*	*	23
Cont.	"	rbcL For	rbcL Rev	rcbL	515	*			12

* Primer pair included in the respective sub-pool.

** Probes used for both amplicon 1 and 2.

### Primer pool optimization

**Figure 5 pone-0000924-g005:**
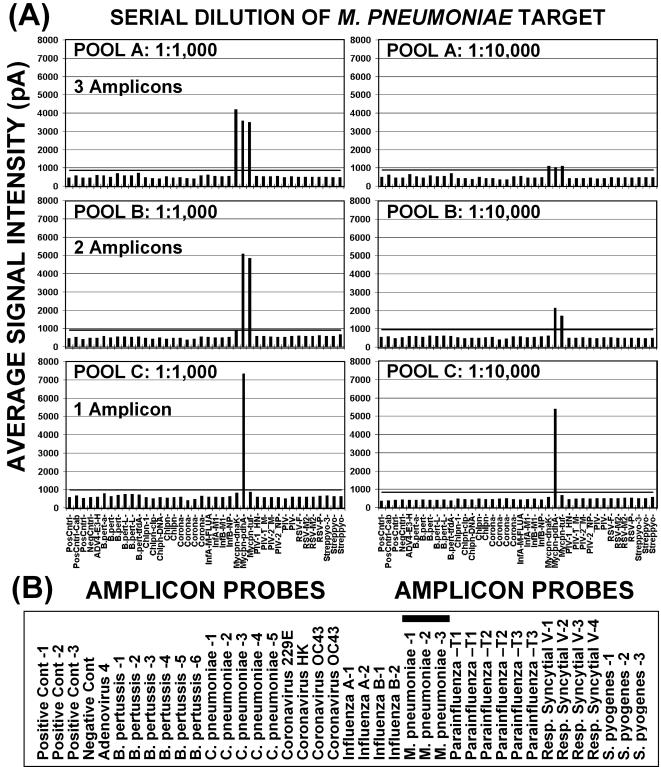
Comparison of assay sensitivity with primer pools 1A, 1B, and 1C. *Mycoplasma pneumoniae* (*M.p*.) DNA, at 1 ng/ul, was serially diluted and amplified with primer pools 1A (3 *M.p*. amplicons), 1B (2 *M.p*. amplicons), and 1C (1 *M.p*. amplicon). Panels in (A) illustrate the sensitivity obtained with the 3 pools at *M.p*. DNA dilutions of 1∶1,000 (left) and 1∶10,000 (right). Average signal intensities in picoamps for each set of amplicon probes (vertical bars) are shown at the left. The horizontal bar within graphs indicates the assay cutoff (mean 3±SDM). The PCR amplicons analyzed for each pathogen are listed in panel (B) and the three *M.p*. amplicons are indicated by thick underline.

Initial studies were conducted with two primer pools consisting of 19 primer pairs for DNA targets and 18 primer pairs for RNA targets (Pools 1A and 2A; Table 1). Subsequent studies to determine primer interactions and optimal target signals on the array resulted in reducing primer pool sizes to increase assay sensitivity (Fig. 5). Primer pool 1B for DNA targets included 10 of the original primer pairs and pool 2B for RNA included 9 of the original primer pairs. A third primer pool for DNA targets, pool 1C, contained only 5 primer pairs (see Table 1). A comparison of DNA target primer pools A, B, and C, shown in Fig. 5, demonstrates the increased sensitivity of pools 1B and 1C over pool 1A in detecting increasing dilutions *M. pneumoniae* DNA. All pools produced sufficient PCR products for detection on arrays at a 1∶1,000 dilution of target DNA (1 pg). Pools B and C were effective on samples diluted 1∶10,000; but only pool C was effective at dilutions above 1∶10,000.

Primers for assay positive control *A. thaliana* RNA and DNA RT-PCR reaction spike-ins, originally consisted of three primer pairs: 1) A. thaliana RCA gene primers for DNA targets, 2) *A. thaliana* Cab gene primers for RNA targets, and 3) *A.thaliana* rcbL gene primers for both DNA and RNA targets. The primer set for the rcbL gene was subsequently removed from the final assay to improve performance.

### Sensitivity and specificity studies

**Figure 6 pone-0000924-g006:**
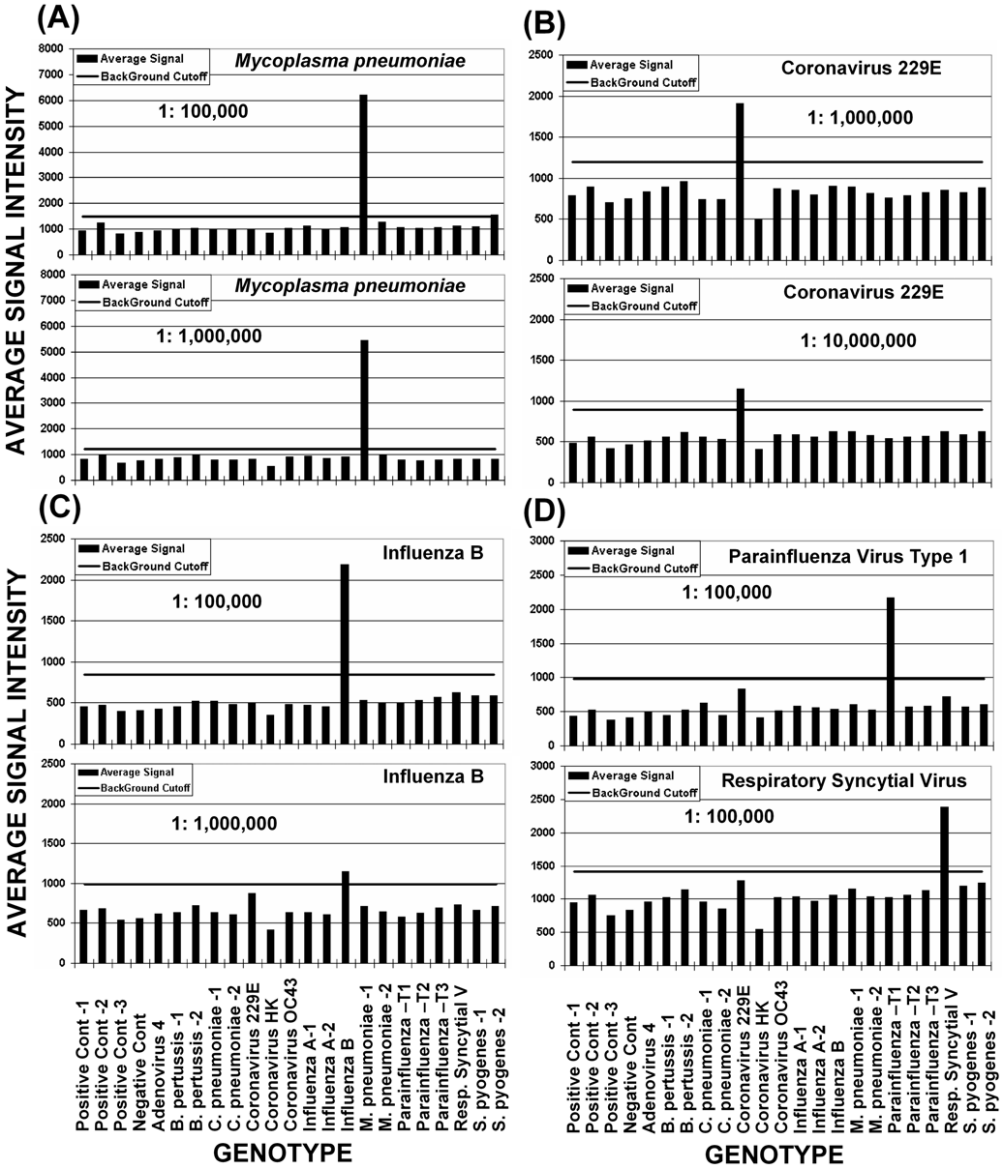
Results of sensitivity studies for *Mycoplasma pneumoniae* (A); coronavirus 229E (B); influenza B (C); and PIV 1, and RSV (D). Vertical bars indicate the mean signal intensities in picoamps for positive genotypes (above cutoff bar) and negative genotypes (below cutoff bar). Horizontal bars indicate the assay cutoff, which is based on the mean of the negative control probes, plus 3 standard deviations of the mean. Relative probe electrochemical signal intensities in picoamps are shown to the left and probe identities are shown below. The identity of the pathogen tested and the dilution factor are shown on each graph. Beginning target nucleic acid concentrations are shown in Table 2.

**Figure 7:**
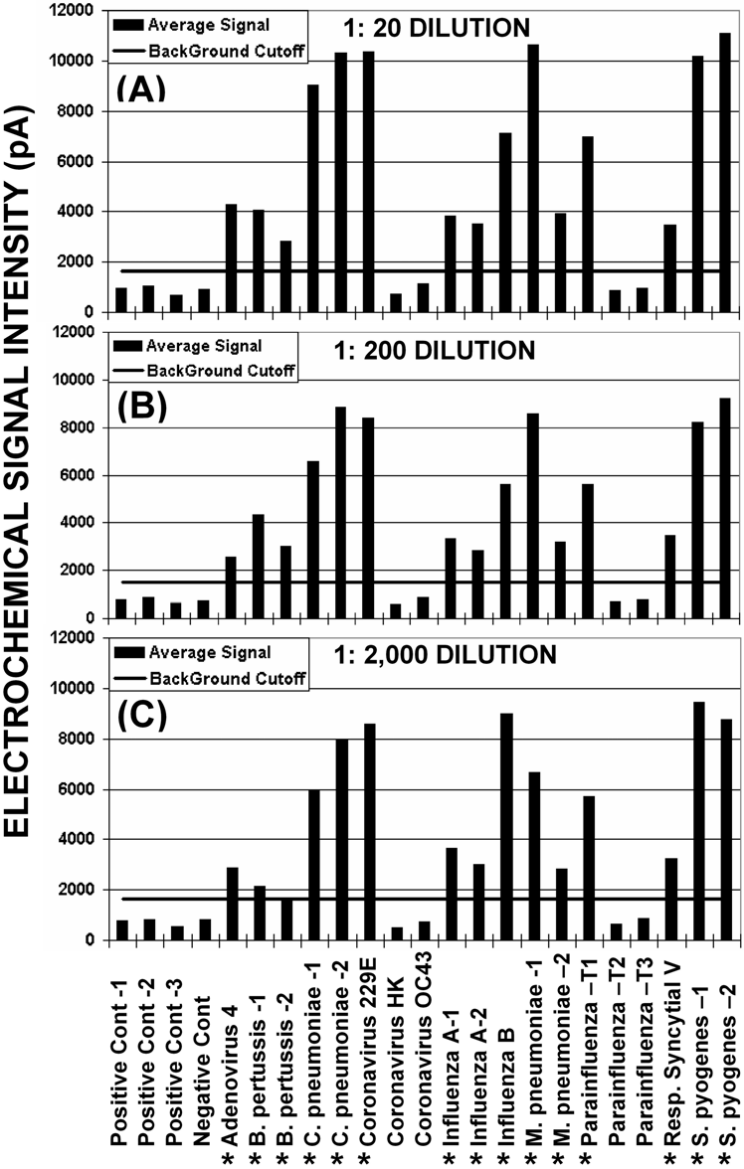
Example of sensitivity and specificity tests for multiple-organism assays. Ten genotypes of interest (adenovirus 4, *B. pertussis*, *C. pneumoniae*, coronavirus 229E, influenza A, influenza B, *M. pneumoniae*, parainfluenza Type 1, respiratory syncytial virus, and *S. pyogenes*) were simultaneous amplified in two PCR reactions (5 DNA and 5 RNA targets) that were combined and hybridized on the same chip. Assays were run with pathogen nucleic acid dilutions of: (A) 1 to 20 (1∶20), (B) 1 to 200 (1∶200), and (C) 1 to 2,000 (1∶2000) that were based on the sample concentrations listed in Table 2. Vertical bars indicate average genotype probe signals in picoamps and horizontal bars indicate the assay cutoff (mean of negative control probes plus three standard deviations of the mean). Asterisks indicate the 10 target genomes that were amplified.

Using optimized primer pools (1C and 2B) and probe sequences, sensitivity studies were conducted on 10-fold serial dilutions of NA from each target organism. Starting NA concentrations were determined spectrophotometrically and from vendor specifications (Table 2). Exact concentrations of specific target organism NA was probably less because of host cell NA contamination and loss of material during NA isolation. In addition, when NA concentrations were based on PFU/CFU, the contribution of non-viable organism NA could not be determined. The URI genotyping assay could detect NA dilutions to approximately 1 to 10 genomes for 9 of 12 target categories (Table 2 and Figure 6). Less sensitivity was achieved for *Bordetella pertussis* and parainfluenza (PIV) types 1 and 3. The URI genotyping assay was also tested by including NA from the 5 organisms with DNA genomes and the 5 organisms with RNA genomes in separate PCR reactions, and then combining reactions for hybridization to arrays (Fig. 7). This did not result in the loss of a specific pathogen signal for the dilutions tested.

**Table 2:** Assay sensitivity tests for viral and bacterial URI pathogens.

Sample	PFU-CFU/Reaction	Conc. ng/ul	Highest Dilution	Genomes Detected a	DNA-b RNA/Rx
Adenovirus 4	4,375^a^	90	1∶10,000	1	10.0 pg
*B. pertussis*	4,287,000	15	1∶10,000	429	3.0 pg
*C. pneumoniae*	438^a^	116	1∶100,000	1	2.3 pg
Coronavirus 229E	4,375^a^	128	1∶10,000,000	1	25.6 fg
Coronavirus HK	-c	-	-	-	-
Coronavirus OC43	43,750^a^	760	1∶10,000	5	152 pg
Influenza A	?	4.7	1∶100,000	(1)	90 fg
Influenza B	43,750^a^	44.7	1∶1,000,000	1	89 fg
*M. pneumoniae*	1,393,322	1	1∶1,000,000	1	2 fg
Parainfluenza Type 1	4,375,000^a^	512	1∶100,000	44	10 pg
Parainfluenza Type 2	-	-	-	-	-
Parainfluenza Type 3	43,750,000^a^	40	1∶100,000	438	800 fg
Resp. Syncytial virus	4,375^a^	37	1∶100,000	1	740 fg
*S. pyogenes*	9,775,345	16	1∶1,000,000	10	32 fg

a Based on culture titers provided by ATCC.

b Based on nucleic acid concentrations.

c Not tested (included for specificity).

Signal specificity was determined by comparison of specific target signal to negative control probe signal and to signal from non-target probes. As shown in Figure 4A and B, very low signal (background) was seen on probes that were not specifically targeted. Probes for coronavirus and PIV represented three subtypes of each virus: coronavirus 229E, HK, and OC43; and PIV types 1, 2, and 3. When one subgroup was targeted, little signal was seen on probes for the other two subgroups as shown in Figures 6B, 6D and 7.

## Discussion

A variety of multiplexed assays for the identification of mixed targets have been reported that use electrophoresis, molecular beacons, or enzymatic assays for detection [8], [37], [38]. Solid-phase PCR on microarrays has also been used for genotyping assays [39], [40]. This approach allows for either specific array probe extension and labeling for genome identification or on-chip isothermal, strand-displacement amplification. An assay combining a multiplexed RT-PCR platform with a library of photocleavable Masscode tags that are identified by mass spectrometry has been used to identify 22 respiratory pathogens [41]. Lin and colleagues [42] have used multiplexed RT-PCR combined with resequencing microarrays to identify multiple respiratory tract pathogens in a 12-hour assay. The majority of these approaches either do not provide sequence information, utilize expensive equipment that cannot be easily transported, require a high level of expertise to operate equipment or are not easily modified for additional pathogens.

In this study, we describe a rapid and sensitive genotyping assay for URI. This genotyping assay uses the same protocol to detect both bacteria and viruses that produce upper respiratory clinical symptoms; and it relies on the simultaneous examination of numerous loci to differentiate one organism from another. Microarray technologies can provide a significant amount of information at disparate loci in a highly redundant fashion that is helpful when discriminating between similar or different organisms or compensate for cases where genomic variation can result in false negative signals. In this array design, probes were developed to be species and sub-species-specific and then crosschecked against databases to minimize the possibility of cross-hybridization of target with other probes or with contaminating host NA. Multiple probes per pathogen (approximately 30 to 100) were used to overcome potential variability in pathogen genomes and also to provide sequence information at disparate loci. PCR primers were designed to flank the conserved regions that contained multiple complementary probe sequences. They also were designed such that the one-tube RT-PCR reaction produced biotin-labeled, single-strand target for hybridization to arrays. Decreasing the number of primer pairs per multiplex RT-PCR also increased assay sensitivity. Greatest sensitivity was achieved with one primer pair per genome of interest.

Equal labeling for GC-rich and AT-rich genomes was promoted by including both biotin-dCTP and biotin-dATP in PCR reactions. This is especially relevant for *B. pertussis* with a genome GC content of 67% and RSV with a GC content of 33%. Greater assay sensitivity was obtained by using 5′ biotinylated reverse primers in place of biotin-dCTP incorporation during amplification, as biotin-dCTP inhibits the PCR reactions (data not shown). However, with this increase in sensitivity, we also observed a slightly higher background signal and also a specific pattern of binding of free biotinylated primers to some probes. Removing microarray probes that bind to the free biotinylated primers can reduce this background artifact.

The arrays were validated by hybridizing labeled target that was generated from RNA or DNA from all viruses and bacteria represented on the upper respiratory chip, which includes influenza A and B; parainfluenza types 1, 2, and 3; adenovirus 4; respiratory syncytial virus; coronavirus 229E, HK, and OC43; *Streptococcus pyogenes; Bordetella pertussis; Chlamydia pneumoniae; and Mycoplasma pneumonia*. The arrays contain multiple probes per amplified region and multiple regions per organism that correspond to key distinguishing elements of each organism or subtype. However, to increase assay sensitivity, the final primer sets contained only one amplicon per organism. All virus and bacteria samples could be identified individually or in mixtures of 10 organisms within 5 hrs. Under optimal conditions, the assay was also sensitive, detecting 1 to 10 genomes, in most cases; and specific, detecting signal predominately on the pathogen probes of interest and not on probes for closely related species or serotypes as shown in Figures 4 through [Fig pone-0000924-g005] [Fig pone-0000924-g006] 7.

Studies to determine assay sensitivity were, in many cases, difficult to analyze because of complexities in quantifying target nucleic acid in a mixture with host nucleic acid. Most of the stock target bacterial DNA and viral RNA concentrations used in this study were based on PFU/CFU estimates provided by ATCC. Virus PFU were determined from cell culture lysates that would contain host cell nucleic acid. Given that one human cell contains approximately 7 pg of DNA, host cell nucleic acid contamination of samples could be very high. Thus, final results of assay sensitivity, based on PFU, could be underestimated in some cases.

In this paper, we demonstrate the usefulness of ECD and microchips as a diagnostic platform for genotyping URI. In addition, this type of microarray can easily be subdivided into multiple sub-arrays; e.g., 4 sectors of 2240 electrode features per array, which allows multiple assays on one array and provides significant cost savings. Also, because of the platform's flexibility, the array content and primer pools can easily be changed to reflect the needs of clinicians in different localities and different seasons. The high manufacturability of semiconductors and their adaptability to inexpensive electrochemical microarray readers, which are small and field deployable, make these assays much more powerful than simple Real Time-PCR systems. In essence, the array provides a much higher level of confidence in a result akin to DNA sequencing; and, in addition, upfront bioinformatics and judicious choice of probes and loci allow a greatly simplified and speedier analysis of complex organisms or mixtures thereof. Ghindilis et al. [35] have described electrochemical detection and the application of this technology on the CombiMatrix array platform. This platform is able to provide very similar sensitivity and reproducibility to state of the art fluorescent detection. Their analysis of data correlation between the electrochemical detection and standard fluorescent detection techniques demonstrated that the array-to-array correlation for both detection techniques is very high with an average correlation coefficient of 0.94 [35].

The use of multiplexed RT-PCR combined with electrochemical detection on micro-arrays, as described here, has several advantages over traditional assays, including RT-PCR alone: 1) The ElectraSense® ECD scanner is small, portable, and rugged and can easily be automated or incorporated as a “sub-system” into other genomic analysis systems; 2) The ECD scanner is faster, easier to use, and less expensive than fluorescence scanners; 3) Multiplexing is simpler because the array sorts out multiple signals that can arise from miss-priming in complex samples; 4) There is no need to sequence PCR products because the binding of multiple probes to specific targets provides sequence-like information; 5) This format is flexible, allowing for multiple probes, selection of the most reactive probes through iterative tests, and thus increased probability of making correct calls at low target concentrations and with rare isolates/subtypes. This platform also allows one to easily include the detection of single nucleotide polymorphisms and short stretches of sequence within an assay; and 6) A low level of technical expertise is needed to run assays and analyze data.

Disadvantages to using PCR for amplification are the possibility of contaminating reactions with amplicons from previous assays and amplifying genetic material from potential pathogens that are part of the normal flora. We are addressing amplicon contamination by developing a cartridge format for amplification, labeling, and hybridization of target. This cartridge contains the amplicons and is disposed of after analysis [36]. With respect to amplifying genetic material from pathogens that are part of the normal flora, PCR amplification is highly sensitive and is only qualitative at best. Therefore, the assay may (as with any standard bacteriological test) report the presence of pathogens in the absence of establishing a direct correlation with disease. As reported by Konno et al. [42], the flora of a healthy individual can contain pathogenic species (e.g., *S. pyogenes, S. pneumoniae, H. influenzae* and *Moraxella catarrhalis*). They detected these organisms in a group of healthy 0 to 6 year olds at approximately the same rate as in patients with acute upper respiratory infections. These organisms were also found in older healthy subjects (7 to 74 year olds) but at lower levels [43]; and they were also recovered at a higher rate in smokers than nonsmokers [44]. Clinicians must therefore verify the results of highly sensitive assays with patient symptoms that would suggest a bacterial infection. The inclusion of bacterial genetic markers for antibiotic resistance or virulence in microarray assays would also help guide the clinician in establishing an accurate diagnosis and appropriate treatment regimen.

Rapid identification of upper respiratory pathogens will significantly decrease the time and cost for the identification of potential lethal virus and bacterial strains and lead to better treatment and management of infections. Microarray and biosensor technologies show great promise for virus and bacteria detection and genotyping and are needed for rapid effective treatment, environmental monitoring and the detection of bioterrorism agents [22], [29]. Advances in microarray processing and simpler detection described here provide a path to migration of microarray technologies to clinical applications.

## Materials and Methods

### Viral and bacterial samples

Viral and bacterial samples were obtained from ATCC either as cultures (Influenza B: VR-823, PIV-1: VR-94, PIV-3: VR-93, Coronavirus OC43: VR-1558, Coronavirus 229E: VR-740, RSV-B: VR-1580, Adenovirus 4: VR-1572, and *Chlamydia pneumoniae*: VR-1355) or genomic DNA (*Mycoplasma pneumoniae*: 15531D). Dr. Wanda Lyon provided nucleic acid (NA) for additional samples, including Influenza A, *Bordetella pertussis*, and *Streptococcus pyogenes*. Viral RNA was extracted from clinical samples and archived cultures with an RNAeasy kit (Qiagen, Chatsworth, CA) or from active cultures (ATCC, Manassas, VA) with Trizol (Invitrogen, Carlsbad, CA) following the manufacturer's protocol and precipitated with ethanol and sodium acetate (Sigma, St. Louis, MO) in the presence of yeast tRNA (Invitrogen) and glycogen (Ambion, Foster City, CA). Genomic DNA from bacterial cultures was isolated with Qiagen DNeasy columns. *Chlamydia pneumoniae* DNA was isolated by resuspending the culture pellet in 475 µl Tris-EDTA, adding 25 µl 10% SDS (Sigma) and 2 µl or 20 mg/ml proteinase K (New England Biolabs, Beverly, MA) and incubating over night at 37°C. After heat inactivation at 70°C for 20 min, DNA was extracted once with phenyl/chloroform/isoamyl alcohol (25∶24∶1; Sigma), twice with chloroform (Sigma), and precipitated with 3M sodium acetate and ethanol. Concentrations of RNA and DNA were measured with a NanoDrop ND-1000 Spectrophotometer (NanoDrop Technologies, Wilmington, DE). Positive control Arabidopsis DNA (RCA at 1 ng/µl) and RNA (CAP at 1 ng/µl) for RT-PCR reaction verification was obtained from Invitrogen (SpotReport-3 Array Validation System). Positive control RNA and DNA were diluted (1∶20) in a solution of 20 µl yeast tRNA (1 mg/ml; Invitrogen) and 4.8 µl BSA (10 mg/ml; New England Biolabs) in 475.2 µl 10 mM Tris pH 7.4 (dilution buffer) (Sigma).

### Microarray, probe and primer design

Microarrays used in this study were ElectraSense® 12K microarrays (CombiMatrix Corp. Mukilteo, WA) that contain 12,544 individually addressable electrodes linked by the semiconductor circuitry. Contact pads on the array allow for electrical connectivity with an external device to control custom synthesis of unique probes at each electrode, and electrochemical detection with the ElectraSense® microarray reader (CombiMatrix Corp). Each electrode has a distinct DNA probe above it, and each electrode can be read electronically or fluorescently to determine the level of hybridization for a specific DNA sequence.

Probe and primer design was achieved by aligning URI pathogen sequences obtained from GenBank and finding suitable regions for amplification and detection of each organism. A context database was then created for each organism to be typed. This was accomplished by collecting all available genomic sequences sharing the same genus or serotype as the sequence being investigated. Next, a structured BLAST database was created by incorporating genus and species information into the label of each context genomic sequence. For each organism to be typed, the gene sequences were extracted from the genomic record using the GenBank annotation. These gene sequences were then searched against the context database. All the matches between a gene of interest and a given context database genome were parsed to reflect each gene's conservation within that related genome. The specificity of each gene was reported in relation to the context database.

Genes were chosen for probe design based on their specificity within the organism's home clade. A combination of sub-type-specific, species-specific as well as clade-specific genes were chosen. The desired specificity for gene choice depended on what one wanted to discern within the topography of the organism's home-clade. In general, for each organism of interest, genes were chosen whose specificities included unique as well as conserved sequences. If discernment was to be made between two closely related organisms, more specific genes that would allow discrimination were chosen. Five to ten genes were initially selected for each organism of interest. For each gene chosen, isothermal Tm 60°C probes were designed in half-probe-length steps across each sequence. Only good-quality probes, as defined by convention, were accepted. For example, probes could not contain repeat region greater than 6 bp in length, significant secondary structure, or GC percentage less than 35% or greater than 65%.

After design, the probes were searched against the context database and their specificity was reported with respect to the context database. Regions within genomes that had the desired specificity were then selected. These regions were approximately 500 bp in length, and contained the probes that exhibited the desired specificity. The first and last probes of each region were designated as amplifying primers (the reverse primer was anti-sense), and the sequences in between were used as probes on the microarray. Where possible, PCR primers were chosen that were conserved within the clade. The forward primers were created with a Tm of approximately 50°C where as reverse primers were created with a Tm of approximately 65°C. This differential in temperature allowed for both amplification and single-strand target production. Bacterial and viral genes used for probe and primer selection are listed in Table 1.

### Target amplification and labeling

Two methods were used for labeling NA during RT-PCR: biotin-14-dCTP incorporation (see Fig. 1) or biotinylated reverse primers (Integrated DNA Technologies, Coralville, IA). Twenty-five µl reaction mixes included 12.5 µl of reaction buffer (Invitrogen; SuperScript III One-Step RT-PCR kit with Platinum Taq), 2 µl of 5 mM MgSO4, 0.7 µl of 0.4 mM biotin-14-dCTP (Invitrogen) or biotin-11-dATP (PerkinElmer, Boston, MA), 2 µl primer pool (IDT, Coralville, IA), 1 µl *Arabidopsis* positive control mix, 0.5 µl enzyme mix, 2 µl RNA or DNA sample (diluted in positive control dilution mix (see above) and 4.3 µl dH2O. Thermal cycling parameters were: (50°C–30 min)×1 cycle; (94°C–3 min)×1 cycle; (94°C–30 sec, 56°C–45 sec, 72°C–45 sec)×40 cycles; (94°C–30 sec, 68°C–60 sec)×30 cycles and (72°C–5 min)×1 cycle. Amplification product was initially identified by loading 3.0 µl of each reaction product on 6% polyacrylamide gels (Invitrogen) and staining with SYBR Green I (Molecular Probes, Invitrogen). For ECD assay sensitivity, 10-fold serial dilutions of NA samples (10-1 to 10-8) were made in buffer containing 40.0 µg yeast tRNA (Invitrogen) and 9.6 µg BSA (NEB) per 1.0 ml of dH2O.

### PCR optimization

Each PCR reaction component, including magnesium, biotin and primer pool concentration, was tested individually at various concentrations. Primer pairs were tested individually with their respective template, and each primer sequence was tested “in silico” (Clone Manager Suite, Version 7.1, Scientific and Educational Software Durham, NC) for interactions with multiplexed primers to eliminate cross reactivity. PCR temperatures and dwell times were adjusted for optimal sensitivity. The composition of primer pools was tested by varying the number of primer sets for each target organism (see Table 1).

### Microarray hybridization and electrochemical detection

**Figure 8:**
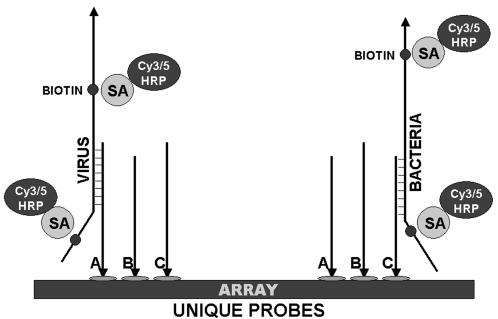
Hybridization and detection of viral and bacterial targets on microarrays. Biotinylated, single-strand target is hybridized to complementary probes on the microarray (A, B and C) and labeled by the addition of streptavidin (SA)-horseradish peroxidase (HRP) (for electrochemical detection) or streptavidin-Cy3/Cy5 (for fluorescent detection).

Initially, microarrays were incubated for 30 min at 45°C in 50 µl of a solution consisting of 5 ml of 2×hybridization solution (see below), 1 ml of 50×Denhardt's solution (Sigma) and 0.5 ml of 1% SDS (Sigma). For hybridization (see Fig. 8), PCR reactions from primer pools were combined and mixed 1∶1 with 2×hybridization buffer, which consisted of 6 ml of 20×SSPE (Ambion, Austin, TX), 0.1 ml of 10% Tween 20 (Sigma), 0.56 ml of 0.5 M EDTA (Ambion), 0.5 ml of 1% SDS (Sigma) and 3.84 ml of dH2O (Ambion). Microarray hybridization chambers were filled (50 µl volume) and sealed with tape. The arrays were incubated for 1 hr at 45°C with rotation in a hybridization oven (Fisher Scientific, Pittsburgh, PA) and washed for 5 min at 45°C with 3× SSPE with 0.05% Tween 20; twice with 2×PBS with 0.1% Tween 20 (PBST); and then blocked for 5 min with 5×PBS/Casein (BioFX Laboratories, Owing Mills, MD). For labeling, microarrays were incubated for 30 min with ExtrAvidin Peroxidase (Sigma) diluted 1∶1000 in BSA Peroxidase Stabilizer (BioFX). Arrays were washed twice with 2×PBST, and twice with pH4 Conductivity Buffer Substrate (BioFX). TMB Conductivity 1 Component HRP Microwell Substrate (BioFX) was added to the array, and it was scanned immediately with an ElectraSense® microarray reader (CombiMatrix Corp). This instrument measures μA at each of 12,544 electrodes on the array in 25 sec and outputs data in picoamps to a simple text file that can be used to create a pseudoimage or can be transferred to and graphed with an Excel macro.

### Data analysis

For the analysis of raw intensity data from arrays, we developed a simple sorting and averaging algorithm in Excel. The organization of the data was keyed to the probe name. (e.g.: Case 1) PosCntrl-ATRCA|Probe-031; Case 2) PosCntrl-Cab|Probe-004; Case 3) Corona-229E|Probe-019; and Case 4) Corona-HK|Probe-001). Probe sequence information was organized and stored according to organism type and sub-type; with the sub-type section of the probe name occurring before the main delimiter, the bar (“|”), and the type section of the probe name appearing before the dash (“-“), when present. Thus the types for Cases 1 and 2 are “PosCntrl”, and the sub-type for Cases 3 and 4 are “Corona-229E” and “Corona-HK”, respectively. The data could be further divided into individual amplicon-specific probes. Once broken down into their groupings, the average signal for each type and sub-type was calculated, presented and graphed as a simple bar graph in picoamps. For an average signal to be considered significant, it had to be greater than three standard deviations above the mean signal of the negative controls. This calculation is termed the Assay Cut-off and is graphed as a horizontal line along the bar graph for simplicity of presentation and interpretation. Through this method of analysis, multiple positive results may be detected in a single assay.
